# Adherence to the Gluten-Free Diet during the Lockdown for COVID-19 Pandemic: A Web-Based Survey of Italian Subjects with Celiac Disease

**DOI:** 10.3390/nu12113467

**Published:** 2020-11-12

**Authors:** Alice Monzani, Elena Lionetti, Enrico Felici, Lucia Fransos, Danila Azzolina, Ivana Rabbone, Carlo Catassi

**Affiliations:** 1 Division of Pediatrics, Department of Health Sciences, Università del Piemonte Orientale, 28100 Novara, Italy; ivana.rabbone@med.uniupo.it; 2 Department of Pediatrics, Marche Polytechnic University, 60020 Ancona, Italy; m.e.lionetti@staff.univpm.it (E.L.); c.catassi@staff.univpm.it (C.C.); 3 Pediatric and Pediatric Emergency Unit, Children Hospital, 15121 Alessandria, Italy; enrico.felici@ospedale.al.it; 4 Piedmont Section, Italian Celiac Association, 10136 Turin, Italy; fransoslucia@hotmail.com; 5 Department of Translational Medicine, Unit of Medical Statistics and Cancer Epidemiology, Università del Piemonte Orientale, 28100 Novara, Italy; danila.azzolina@uniupo.it

**Keywords:** celiac disease, gluten-free diet, COVID-19, coronavirus, lockdown, diet compliance, web survey, telemedicine

## Abstract

We aimed to assess the perceived impact of the lockdown, imposed to control the spreading of COVID-19, on the adherence of Italian celiac disease (CD) subjects to the gluten-free diet by a web-based survey. A total of 1983 responses were analyzed, 1614 (81.4%) by CD adults and 369 (18.6%) by parents/caregivers of CD children/adolescents. The compliance with the GFD was unchanged for 69% of the adults and 70% of the children, and improved for 29% of both. The factors increasing the probability to report stricter compliance were the presence of CD symptoms in the last year before the lockdown (odds ratio (OR) 1.98, 95% confidence interval (CI) 1.46–2.26), a partial usual adherence to gluten-free diet (GFD) (OR 1.91, 95% CI 1.2–3.06), and having tried recipes with naturally gluten-free ingredients more than usual (OR 1.58, 95% CI 1.28–1.96) for adults; the presence of CD symptoms in the last year (OR 2.05, 95% CI 1.21–3.47), still positive CD antibodies (OR 1.89, 95% CI 1.14–3.13), and other family members with CD (OR 2.24, 95% CI 1.3–3.85) for children/adolescents. Therefore, the lockdown led to a reported improved adherence to the GFD in one-third of the respondents, in particular in those with previous worse disease control, offering the opportunity to avoid sources of contamination/transgression and increase the use of naturally gluten-free products.

## 1. Introduction

In December 2019, a cluster of pneumonia cases caused by a novel coronavirus, named SARS-nCoV-2, occurred in Wuhan, China, and rapidly spread initially in Europe and later throughout the world. On 11 March, 2020, coronavirus disease 2019 (COVID-19) was declared a pandemic by the World Health Organization [1]. On 9 March, 2020, the Italian government imposed a national lockdown, and in the whole Country the same restrictive measures for the movements of the population were introduced with the aim of controlling the viral spreading. From then until 18 May, the Italian population was forced into an unprecedented homebound isolation. This has indeed impacted people’s lifestyle and sometimes modified their quality of life. Such changes have been even more relevant for people with chronic diseases. Because of the COVID-19 pandemic, healthcare providers have abruptly changed their care delivery to protect patients and staff from infection and to reallocate the resource towards the greatest acute needs. Elective, routine, and nonemergency casework has stopped, and treating or supporting people with non-urgent and long-term conditions at a distance from healthcare providers by telemedicine and eHealth approaches has become imperative [2].

In the gastroenterological area, the impact of the lockdown on celiac disease (CD) has only limitedly been addressed [3]. CD has the peculiarity of merely dietetic treatment, which could be impacted in different ways by the restrictive measures, possibly implying difficulties in the supply of gluten-free products [3] or avoiding eating out, known to be an occasion of potential contamination or transgression to the GFD [4,5]. Previous findings on the impact of the lockdown on dietary habits in the general population are controversial, with some studies reporting a decrease in nutritional quality of diet on average worldwide [6,7,8,9,10,11,12], while a recent survey showed an increased healthy eating during the pandemic, thanks to less eating out and increased cooking [13]. Therefore, we aimed to assess the impact of the restrictive measures on the adherence of Italian CD subjects to the gluten-free diet (GFD) and on the management of the disease during the lockdown, by a web-based survey.

## 2. Materials and Methods

### 2.1. Study Design

We performed a cross-sectional survey, conducted in Italy during the lockdown by an online questionnaire, self-administered in Italian language, according to the Checklist for Reporting Results of Internet E-Surveys (CHERRIES) [14].

The probability sample is considered demographically representative of the whole population. An information sheet as the first page of the online survey was set, with participants required to check a box to indicate consent before accessing the survey. All the potential participants were fully informed about the study, the reason for conducting the research, how the data will be used, the extent of privacy, anonymity, and confidentiality, the voluntary nature of participating, information in case the respondents changed their mind during the survey, along with contact details for further information. The participation in the survey was voluntary and anonymous and took about 10–15 min. Confidentiality of the information was ensured, and no financial incentive to participate in the study was offered. The data were protected by a password which only the Principal Investigator had access to. The study was approved by the local Ethics Committee (CE 83/20).

### 2.2. Survey Participants

The web-based survey addressed both adult subjects (aged ≥18 years) with a certified diagnosis of CD and parents/caregivers of children/adolescents (aged <18 years) with a certified diagnosis of CD.

### 2.3. Survey Dissemination

The Italian Celiac Society distributed to all members a link to the survey by their mailing list. The link to the web-based survey was posted also on chats through freely communicating apps (WhatsApp), and social networks (Instagram and Facebook), five weeks after the beginning of the lockdown (from 29 April to 1 June, 2020). Furthermore, snowball sampling, where existing study subjects recruit future subjects from among their acquaintances, was also used, as respondents were encouraged to pass it on to others. Individuals were directed via an electronic link to an online survey platform (Google Forms).

### 2.4. Variables and Data Sources

At the beginning of the survey, respondents had to select if they were either adults (aged ≥18 years) with a certified diagnosis of celiac disease or parents/care givers of children/adolescents (aged <18 years) with a certified diagnosis of celiac disease. Thus, they were re-directed to the appropriate section. A single respondent could answer both the questionnaires for adult CD subjects and for parents/caregivers of CD children/adolescents. At the end, respondents were able to review and change their answers.

The survey consisted of 33 items, distributed in 4 screens. There was a general part including sociodemographic questions (age, nationality, province of residence, educational level, housing conditions, self-perceived economic status, current employment status, and compliance with restrictive measures) and questions about CD diagnosis, followed by questions addressing different analysis domains (Supplementary Materials S1). The following analysis domains were explored: availability of gluten-free products, adherence to GFD, and access to care for CD during the lockdown. 

Due to the lack of validated questionnaires about this specific topic, the survey was ad hoc designed to assess all the mentioned variables, consistently with the aim of the research. The questionnaire was tested in a sample of voluntary adult CD patients who reviewed the questionnaire individually and provided verbal feedback, also reporting the time needed to fill it.

### 2.5. Study Endpoints

The primary endpoint of this study was the perceived impact of the lockdown on the adherence to GFD of CD subjects. Secondary endpoints included difficulties in finding gluten-free products during the lockdown and the impact of the lockdown on CD management.

### 2.6. Statistical Analysis

Only completed questionnaires were analyzed. Data were summarized according to groups as median and interquartile range (IQR, 25th–75th percentile) and analyzed using the Wilcoxon test. Categorical variables, whenever dichotomous or nominal, were reported as frequencies and percentages and analyzed through the Chi-square test. Univariable analysis and multivariable proportional odds ordinal logistic regression were carried out to quantify the effects of the covariates on the different degrees of compliance to GFD. The estimated Odds Ratios (OR) together with the 95% confidence intervals have been reported.

The statistical analyses were conducted using R 3.5.2 [15] with the rms packages (Regression Modeling Strategies. R package version 4.1-3. 2014). The threshold of statistical significance was 0.05 for all tests used (two-tailed).

## 3. Results

### 3.1. Study Population

**Figure 1:**
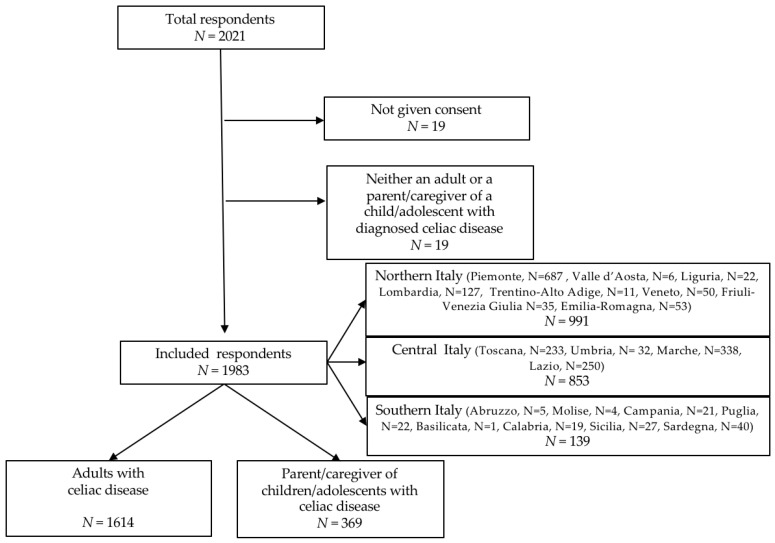
Flow diagram of the survey.

We received a total of 2021 responses; 19 (0.9%) subjects did not give their consent to the interview, and 17 (0.8%) were excluded because they declared not to be either adults with a certified diagnosis of CD or parents/caregivers of children/adolescents with CD. Therefore, 1983 responses were analyzed. Out of them, 1614 (81.4%) were filled in by adults with CD and 369 (18.6%) by parents/caregivers of children/adolescents with CD. The flow diagram of the survey is shown in Figure 1.

The response rate for all the items was higher than 93%. Overall, respondents were from 103 out of 110 Italian provinces: 50% from Northern Italy, 43% from Central Italy, 7% from Southern Italy. Among both adult subjects with CD and parents/caregivers of CD children, more than 99% were Italian (1607/1614 and 366/369, respectively). The median (IQR) age was 39 (28–50) years in the group of adults with CD and 45 (41–49) years in the group of parents/caregivers of CD children/adolescents. The median (IQR) age of the children whose parent/caregiver took part in the survey was 12 (7–14) years.

### 3.2. Demographic and Socio-Economic Characteristics

The baseline demographics and socio-economic features of the respondents are described in Table 1.

More than half of the respondents in both groups had an education degree lower than bachelors, the majority of the overall respondents were employed and were not going to the workplace during the lockdown. In most cases, their household members during the lockdown were unchanged, with no other family members with CD in about three-quarters of the respondents. In both groups, the compliance with the restrictive measures given by the Government about lockdown and social distancing was high or very high (97% in adults and 99% in parents/caregivers). Only 1% of the respondents were officially quarantined by the Authorities at the time of the survey. More than half of the respondents reported that the electronic devices in their possession were much or very much adequate to their needs, and about one-third of them considered their income much or very much adequate to their needs. 

### 3.3. CD Diagnosis and Management

The data about CD diagnosis and management are shown in Table 2.

The median (IQR) age at CD diagnosis was 27 (16–39) years in adults and 5 (3–8) in children/adolescents, with a median (IQR) duration of CD of 11 (5–18) and 4 (1–8) years, respectively.

At diagnosis, an upper digestive tract endoscopy was performed in 89% of the adults and in 39% of the children/adolescents, and the diagnosis was made because of symptoms suggestive for CD in 75% and 73% of cases, respectively. At the last follow-up control, 76% of the adults and 65% of the children tested negative for CD antibodies. In the last year, before the COVID-19 pandemic, 33% of the adults and 27% of the children had experienced symptoms deemed attributable to CD. As for the usual compliance with GFD, 94% of the CD adults and 98% of the parents/caregivers of CD children reported strict compliance.

### 3.4. GFD during the Lockdown

The data about the GFD during the lockdown are reported in Table 3.

About one-third of the respondents reported that they had difficulties in finding gluten-free commercially made products dispensed with the voucher during the lockdown period, without differences according to the housing condition (both among CD adults, 38% of those living in suburbs/villages vs. 32% of those living in cities, *p* = 0.8, and among parents of CD children, 30% of those living in suburbs/villages vs. 25% of those living in big cities, *p* = 0.2). Moreover, 45% of the adults and 40% of the parents/caregivers reported to have increased the preparation of homemade recipes with naturally gluten-free ingredients (gluten-free cereals, meat, fish, vegetables, fruit, etc.). About 70% of the respondents declared their adherence to the GFD remained unchanged during the lockdown, but 29% of both adults and the parents of children reported an improved compliance, mainly due to not eating away from home and having more time to prepare food. No difference was found in the compliance with GFD according to the first or last weeks of the lockdown.

In the adult group, no difference was found between younger and older subjects with respect to GFD compliance: assuming the median age as the cut-off, increased compliance was reported in 239 subjects (30%) that were <39 year old and in 230 subjects (28%) that were ≥39 year old (*p* = 0.5).

### 3.5. CD Health Care Aspects

The data about CD health care aspects during the lockdown are summarized in Table 4.

During the lockdown, only a small percentage of subjects (16% of adults and 10% of children/adolescents) experienced symptoms attributable to CD, significantly lower than in the last year before the pandemic (*p* < 0.001 for both). Even if about one-third of subjects skipped any previously scheduled appointments related to CD during the lockdown, 90% of adults and 87% of parents/caregivers reported no need for consults for CD-related health care advice, and when needed, advices were provided by telephone or e-mail contact in almost all cases. 

### 3.6. Factors Influencing an Improved Adherence to GFD during Lockdown

Answers from both CD adults and parents/caregivers of CD children were analyzed according to their reported adherence to GFD during the lockdown, and the significant ones are shown in Table 5 and Table 6.

As shown in Table 5, in adult CD subjects, stricter compliance with GFD during the lockdown was more likely reported by those experiencing CD symptoms in the last year and during the lockdown, with a partial usual compliance to GFD, who tried homemade recipes with naturally gluten-free ingredients more than usual, and who reported the lockdown had impacted their management of CD for the better. Conversely, living with three or more cohabitants reduced the probability of stricter compliance during the lockdown. In the multivariable ordinal logistic regression model, only the presence of CD symptoms in the last year before the lockdown (OR 1.98, 95% CI 1.46–2.26), a partial usual adherence to GFD (OR 1.91, 95% CI 1.2–3.06), and having tried homemade recipes with naturally gluten-free ingredients more than usual (OR 1.58, 95% CI 1.28–1.96) increased the probability to report stricter compliance during the lockdown. 

As shown in Table 6, according to what was reported by parents/caregivers, CD children/adolescents who more likely improved their compliance with the GFD were those whose parents/caregivers were not employed during the lockdown, with other family members with CD, with a shorter duration of CD, whose last CD antibodies tested positive, who experienced CD symptoms during the last year and during the lockdown, and whose parents reported the lockdown had impacted the management of CD for the better. In the multivariable ordinal logistic regression model, the presence of CD symptoms in the last year before the lockdown (OR 2.05, 95% CI 1.21–3.47), still positive CD antibodies (OR 1.89, 95% CI 1.14–3.13), and other family members with CD (OR 2.24, 95% CI 1.3–3.85) increased the probability to report an improved compliance during the lockdown.

## 4. Discussion

Our survey is the first to explore the potential effects of the restrictive measure imposed during COVID-19 pandemic on the adherence to GFD and lifestyle of subjects with CD. Likely, CD does not represent itself a high-risk condition for the development of severe forms of COVID-19 disease and, therefore, CD patients had been reported not to feel more vulnerable because of their CD [3]. Nonetheless, it was foreseeable that lockdown may have impacted their lives differently from healthy subjects, due to both the limitations and the opportunities that the restrictive measures represented in the GFD perspective. Exploring the potential impact of this unprecedented home-bound isolation represents a unique opportunity to assess the management of the GFD in the absence of potential sources of contamination related to social life. In the general population, previous studies on the impact of the lockdown on dietary habits showed conflicting results. Some studies reported a negative worldwide impact on dietary habits by restrictive measures, promoting unhealthy behaviors in both children and adults [6,7,8,9,10,11,12]. Along with the increase in sedentary behaviors, higher odds for elevated frequency of ultraprocessed food consumption (sugary foods, snacks, ready-to-eat frozen foods, and embedded foods) and low consumption of fruit and vegetables were reported [6], resulting in a decrease in nutritional quality of diet on average [7]. However, it has also been reported that the intake of fast food and commercial pastries decreased, while consumption of homemade pastries increased, testifying a significant increase of the cooked-at-home preparations [8,11]. In our survey, the limitations imposed by the restrictive measures seem to have led subjects with CD to virtuous dietary habits. A similar trend was described also by a recent online survey in healthy subjects, showing increased healthy eating during the pandemic, due to less eating out and increased cooking [13].

The most relevant result of our survey was that, despite facing a troublesome and dramatic global situation, almost one-third of the respondents reported a perceived improved adherence to GFD. In most of the subjects, the compliance with the GFD was unchanged compared with the previous period, with only 2% of the adults and 1% of the parents/caregivers reporting worsened compliance. When investigating the causes of the improved compliance, the most reported reason was not eating away from home, both in adults and children/adolescents. It is known that having meals in restaurants/cafes/canteens may represent a potential source of involuntary contamination [16]. Moreover, sharing the meal with non-CD diners could also be the occasion for voluntary transgressions, especially in adolescents [4,5]. Therefore, the forced isolation imposed by the restrictive measure represented an unprecedented condition for lowering the probability of both involuntary and voluntary contamination, leading to a perceived stricter compliance with GFD. Another reason for the improved compliance was reported to be the increased time available for food preparation. Accordingly, more than 40% of the respondents reported to have tried homemade recipes with naturally gluten-free ingredients more than usual, and this was associated with an improved compliance with GFD in adults. The increased cooking using naturally gluten-free ingredients goes hand-to-hand with the difficulties in finding commercially made GFD products, reported by about one-third of the respondents. 

In the minimal percentage of subjects reporting a worse compliance with the GFD, the main reason seems to be the negative feelings related to the COVID-19 period, followed by difficulties in finding commercially made gluten-free products, reported by about one-third of the respondents. Therefore, the reduced compliance could be firstly attributable to depressive feelings and worries for the pandemic situation, and it could be hypothesized that the negative feelings related to the global situation lead someone to a “disinvestment” in his/her own health, resulting in behaviors (lower attention to possible contamination or voluntary transgressions) leading to a worse compliance to the GFD. Moreover, it should be noted that, in Italy, gluten-free products are supplied to patients for free with a voucher only within their region of residence, and the restrictions for people’s movements may have represented a substantial obstacle. In a recent Italian survey, about half of the CD respondents reported to be a little to very much worried about the possible shortness of gluten-free food at the beginning of the pandemic [3]. Along with the difficulties in finding commercially made gluten-free products, the lockdown could have given some CD subjects the chance to discover more possibilities of a strict GFD based on naturally gluten-free ingredients. 

When analyzing in detail which subjects had a higher probability to report stricter compliance during the lockdown, they turned out to be those with CD-attributable symptoms both before and during the lockdown, suggesting a worse disease control. In adults, this inference was supported by the evidence that the reported compliance was improved in those who had only a partial compliance before the pandemic. It could be assumed that a “beneficial” effect of the isolation was higher just in those who were less compliant with the dietetic treatment and that the presence of symptoms prompted them to pay more attention to contamination/transgressions. Actually, the overall prevalence of subjects reporting CD-related symptoms decreased during the lockdown compared with the last year, and the stricter adherence to the GFD experienced during the pandemic by some subjects would possibly lead to a reduction of their symptoms in the following months. Interestingly, both adults and parents/caregivers reporting a higher compliance declared that the lockdown positively impacted their CD management, highlighting the perceived adherence to the GFD as a key element for a satisfactory management of CD.

In children and adolescents, an improved adherence to the GFD was reported when the parents/caregivers were not employed, likely having more time for food preparation, and when there were other family members with CD, suggesting a higher familiarity with the GFD. Children with a more recent diagnosis, especially those with still positive CD antibodies, were more likely to improve their compliance with the GFD.

In addition to the effects regarding the adherence to the GFD, the restrictive measures had an impact on CD management and access to health care consultations. In our sample, most of the respondents did not need consultations for health care advice. This might be explained by the fact that the prevalence of subjects experiencing CD-attributable symptoms significantly decreased during the lockdown. It might be hypothesized that the subjects followed stricter adherence to GFD so as to prevent potential exposure to COVID-19 during hospital/doctor visits if they had disease flare ups due to dietary transgressions. When needed, the most frequently reported way to receive health care advice was remote consultation by telephone or email with family doctors, private doctors, or gastroenterologists. The raising of telemedicine for the management of CD patients during the COVID-19 pandemic has recently been assessed by a two-center Italian survey, pointing out an overall satisfaction of CD subjects for this approach for CD healthcare [3].

The strengths of our study are as follows: (a) it is the first to report on the perception of CD subjects about the impact of the COVID-19-related restrictive measures on the adherence to the GFD; (b) it addresses both CD adults and parents/caregivers of CD children/adolescents; (c) the web-based method gave us the opportunity to reach a geographically dislocated population during a short time. Our study also has some limitations: (a) the lack of use of validated questionnaires; (b) that only the personal perceptions of the respondents are presented, as an intrinsic limit of the surveys. In this specific case, the improved, unchanged, or worsened compliance with the GFD reported by respondents should be verified by CD antibody tests performed after the end of the lockdown, compared with previous ones. Moreover, in the outpatient visits performed after the lockdown it would be interesting to evaluate possible changes in body weight or composition, and to correlate them with lifestyle and dietary habits occurred during the lockdown.

## 5. Conclusions

In conclusion, the global impact of the lockdown on CD was not negative, being the compliance to GFD unchanged for 70% of the respondents, and even improved for 29%, in particular for those with a previous worse disease control, due to reduced occasions for contamination/transgression and increased use of naturally gluten-free ingredients. It is desirable that the positive aspects of dietary management emerged during the lockdown would be maintained also in the future. Nutritionists and gastroenterologists dealing with CD patients should be aware of the virtuous behaviors that have been implemented in this critical period, such as the greater use of naturally gluten-free products, to promote them also in everyday life.

## Figures and Tables

**Table 1:** Socio-demographic characteristics, working and housing conditions of the respondents.

	Adult CD Subjects (*N* = 1614)		Parents/Caregivers of CD Children (*N* = 369)	
Education degree	(*N* = 1610)		(*N* = 369)	
Lower than bachelors		887 (55%)		221 (60%)
Bachelors or higher		723 (45%)		148 (40%)
Occupation	(*N* = 1612)		(*N* = 369)	
Employed		1053 (65%)		295 (80%)
Not employed		559 (35%)		74 (20%)
Current working condition	(*N* = 1613)		(*N* = 346)	
Not working		576 (36%)		145 (42%)
Smart working		647 (40%)		112 (32%)
Going to the workplace		390 (24%)		89 (26%)
Cohabitants	(*N* = 1595)		(*N* = 368)	
none		135 (8%)		2 (1%)
1–2		849 (53%)		79 (21%)
3+		611 (38%)		287 (78%)
Household members during the lockdown	(*N* = 1608)		(*N* = 367)	
Unchanged		1446 (90%)		353 (96%)
Decreased		95 (6%)		6 (2%)
Increased		67 (4%)		8 (2%)
Other family members with CD	(*N* = 1614)		(*N* = 369)	
No		1246 (77%)		291 (79%)
Yes		368 (23%)		78 (21%)
Compliance with the restrictive measures	(*N* = 1614)		(*N* = 369)	
Much/very much		1559 (97%)		367 (99%)
Not at all/little/enough		55 (3%)		2 (1%)
Currently quarantined	(*N* = 1585)		(*N* = 366)	
No		1579 (99%)		362 (99%)
Yes		14 (1%)		4 (1%)
Housing condition	(*N* = 1605)		(*N* = 367)	
Big city downtown		1003 (62%)		181 (49%)
Suburbs/Small–medium town–village		602 (38%)		186 (51%)
Adequacy of electronic devices	(*N* = 1605)		(*N* = 368)	
Much/very much		1097 (68%)		230 (62.5%)
Not at all/little/enough		508 (32%)		138 (37.5%)
Adequacy of the income	(*N* = 1603)		(*N* = 366)	
Much/very much		552 (34%)		103 (28%)
Not at all/little/enough		1051 (66%)		243 (72%)

Data are expressed as number and percentage of the responses for each item. CD: celiac disease.

**Table 2:** Data about CD diagnosis and management.

	Adult CD Subjects (*N* = 1614)		CD Children (*N* = 369)	
Upper digestive tract endoscopy	(*N* = 1614)		(*N* = 369)	
Yes		1579 (98%)		143 (39%)
No		35 (2%)		226 (61%)
Symptoms at diagnosis	(*N* = 1614)		(*N* = 369)	
Yes		1208 (75%)		269 (73%)
No		406 (25%)		100 (27%)
Last CD antibodies negative	(*N* = 1614)		(*N* = 369)	
Yes		1232 (76%)		241 (65%)
No		382 (24%)		128 (35%)
CD symptoms in the last year	(*N* = 1614)		(*N* = 369)	
Yes		532 (33%)		101 (27%)
No		1082 (67%)		268 (73%)
Usual compliance with the GFD	(*N* = 1614)		(*N* = 369)	
Strict		1524 (94%)		360 (98%)
Partial		90 (6%)		9 (2%)

Data are expressed as number and percentage of the responses for each item. CD: celiac disease; GFD: gluten-free diet.

**Table 3:** Data about GFD during the lockdown.

	Adult CD Subjects (*N* = 1614)		Parents/Caregivers of CD Children (*N* = 369)	
Difficulties in finding GFD products	(*N* = 1614)		(*N* = 369)	
No		1088 (67%)		267 (72%)
Yes		526 (33%)		102 (28%)
Homemade recipes with naturally gluten-free ingredients	(*N* = 1614)		(*N* = 369)	
As usual		714 (44%)		190 (51%)
More than usual		720 (45%)		146 (40%)
Never		180 (11%)		33 (9%)
Compliance with GFD during the lockdown	(*N* = 1614)		(*N* = 369)	
Stricter		469 (29%)		108 (29%)
Less strict		29 (2%)		3 (1%)
Unchanged		1116 (69%)		258 (70%)
If stricter, for which reason ^§^	(*N* = 469)		(*N* = 108)	
Not eating away from home		350 (75%)		69 (64%)
More time to prepare food		188 (40%)		46 (43%)
No social life/home alone		12 (3%)		n.a.
Taking better care of myself/my child Higher control over my child		72 (15%) n.a		10 (9%) 28 (26%)
If less strict, for which reason ^§^	(*N* = 29)		(*N* = 3)	
Less time to prepare food		1 (3%)		0 (0%)
Boredom/sadness/worries		14 (48%)		2 (67%)
More occasions for transgression		6 (21%)		0 (0%)
Difficulties in finding gluten-free products		6 (21%)		1 (33%)

^§^ more than one answer can be given to this question. CD: celiac disease; GFD: gluten-free diet; n.a.: not applicable. Data are expressed as number and percentage of the responses for each item.

**Table 4:** Data about CD care aspects during the lockdown.

	Adult CD Subjects (*N* = 1614)		CD Children (*N* = 369)	
CD symptoms during the lockdown	(*N* = 1614)		(*N* = 369)	
No		1356 (84%)		333 (90%)
Yes		258 (16%)		36 (10%)
Consult for health care advice	(*N* = 1519)		(*N* = 356)	
Nobody, no need		1337 (88%)		300 (84%)
Gastroenterologist		41 (3%)		16 (4%)
Dietician/Nutritionist		12 (1%)		2 (1%)
Family/Private doctor		76 (5%)		30 (8%)
Patients association		29 (2%)		4 (1%)
Friends with CD		24 (1%)		4 (1%)
Mode for receiving health care advice	(*N* = 1502)		(*N* = 352)	
No need		1351 (90%)		305 (87%)
Telephone or e-mail contact		139 (9%)		47 (13%)
Outpatient visit		12 (1%)		0 (0%)
Home visit		0 (0%)		0 (0%)
Skipped CD appointments	(*N* = 1588)		(*N* = 366)	
No		1115 (70%)		235 (64%)
Yes		473 (30%)		131 (36%)
Impact of the lockdown on CD management	(*N* = 1614)		(*N* = 369)	
None		1182 (73%)		289 (78%)
For the better		92 (6%)		27 (7%)
For the worse		340 (21%)		53 (14%)

Data are expressed as number and percentage of the responses for each item. CD: celiac disease.

**Table 5:** Factors influencing the adherence to GFD during the lockdown, as reported by adults with CD.

Adherence to GFD		Less Strict	Unchanged	Stricter	Chi2 *p*-Value	OR (95% CI)
Cohabitants	0	2 (7%)	86 (8%)	47 (10%)	0.039	1.19 (0.82–1.74)
	1–2	11 (38%)	578 (52%)	160 (56%)		(REF)
	3+	16 (55%)	440 (40%)	255 (34%)		0.75 (0.6–0.94)
Last CD antibodies negative	Yes	14 (48%)	869 (78%)	349 (74%)	<0.001	(REF)
	No	15 (52%)	247 (22%)	120 (26%)		1.05 (0.82–1.35)
CD symptoms in the last year	No	15 (52%)	804 (72%)	263 (56%)	<0.001	(REF)
	Yes	14 (48%)	312 (28%)	206 (44%)		1.84 (1.47–2.29)
Usual compliance with the GFD	Strict	16 (55%)	1088 (97%)	420 (90%)	<0.001	(REF)
	Partial	13 (45%)	28 (3%)	49 (10%)		2.28 (1.44–3.6)
Homemade recipes with naturally gluten-free ingredients	More than usual	15 (52%)	451 (40%)	254 (54%)	<0.001	1.64 (1.33–2.03)
	As usual/Never	14 (48%)	665 (60%)	215 (46%)		(REF)
CD symptoms during the lockdown	No	20 (69%)	961 (86%)	375 (80%)	<0.001	(REF)
	Yes	9 (31%)	155 (14%)	94 (20%)		1.39 (1.05–1.84)
Impact of the lockdown on CD management	None	15 (52%)	871 (78%)	296 (63%)	<0.001	(REF)
	For the better	0 (0%)	21 (2%)	71 (15%)		9.87 (5.97–16.34)
	For the worse	14 (48%)	224 (20%)	102 (22%)		1.15 (0.88–1.5)

CD: celiac disease; GFD: gluten-free diet; REF: reference value. Total responses for the variable “Cohabitants”: *N* = 1595; for all the other reported variables: *N* = 1614.

**Table 6:** Factors influencing the adherence to GFD during the lockdown, as reported by parents/caregivers of children with CD.

Adherence to GFD		Less Strict	Unchanged	Stricter	Chi2 *p*-Value	OR (95% CI)
Occupation	Employed	2 (76%)	217 (84%)	76 (70%)	0.01	(REF)
	Not employed	1 (33%)	41 (16%)	32 (30%)		2.13 (1.25–3.61)
Other family members with CD	No	3 (100%)	212 (82%)	76 (70%)	0.028	(REF)
	Yes	0 (0%)	46 (18%)	32 (30%)		2 (1.19–3.36)
Years since CD diagnosis ^§^	≤4 yrs	1 (33%)	163 (63%)	43 (40%)	<0.001	(REF)
	>4 yrs	2 (67%)	95 (37%)	65 (60%)		0.47 (0.3–0.74)
Last CD antibodies negative	Yes	0 (0%)	188 (73%)	53 (49%)	<0.001	(REF)
	No	3 (100%)	70 (27%)	55 (51%)		2.44 (1.54–3.87)
CD symptoms in the last year	No	2 (33%)	203 (79%)	64 (59%)	<0.001	(REF)
	Yes	1 (67%)	55 (21%)	44 (41%)		2.31 (1.43–3.75)
CD symptoms during the lockdown	No	1 (33%)	17 (7%)	18 (17%)	0.005	(REF)
	Yes	2 (67%)	241 (93%)	90 (83%)		2.54 (1.26–5.13)
Impact of the lockdown on CD management	None	2 (67%)	215 (83%)	72 (67%)	<0.001	(REF)
	For the better	0 (0%)	10 (4%)	17 (16%)		9.87 (5.97–16.84)
	For the worse	1 (33%)	33 (13%)	19 (18%)		1.15 (0.88–1.5)

CD: celiac disease; REF: reference value. Total responses for all the reported variables: *N* = 369. ^§^ The median value of CD duration was chosen as cut-off to convert the continuous variable “years since CD diagnosis” into a dichotomous variable.

## Supplementary Materials

The following are available online at https://www.mdpi.com/2072-6643/12/11/3467/s1, Questionnaires S1: questionnaires for adult CD subjects and parents/caregivers of CD children.
