# Supplementary material for: Adherence to the Gluten-Free Diet during the Lockdown for COVID-19 Pandemic: A Web-Based Survey of Italian Subjects with Celiac Disease
Source: Nutrients. 2020 Nov 12;12(11):3467. doi: 10.3390/nu12113467 (PMC7698218; doi:10.3390/nu12113467)
Supplement: Supplementary file 1 [file nutrients-12-03467-s001.pdf]

Adherence to a gluten-free diet during the lockdown for COVID-19 pandemic: a web-based survey of Italian subjects with celiac disease – Monzani A. - Online Supplementary Material

<u>Session 1:</u>
Do you agree to take part to the questionnaire?
Yes -> Go to Session 2
No -> End Survey

<u>Session 2:</u>
Please define your condition:
I am an adult (≥18 yrs) with a certified diagnosis of celiac disease -> Go to Session 3
I am a parent/caregiver of a child/adolescent (<18 yrs) with a certified diagnosis of celiac disease -> Go to Session 4

<u>Session 3: Questionnaire for adult subjects (>18 years) with a certified diagnosis of celiac disease</u>

1. Demographic data

- Year of birth
………………..
- Nationality
……………..
- Province of residence
……………..
- Education degree
None
Primary school
Junior High School
High School
Bachelor degree
Master degree
- Are you currently?
Unemployed
Student
Housewife
Employed
Retired
- During these days about my job
I am working from home/smart working
I am regularly going to my workplace
I am going to my workplace less frequently
My job has been suspended
I got fired
I am on maternity leave
I am in layoffs
I am retired
- How many people live in your household?
…………
- Has the number of members in your household changed during the COVID-19 pandemic?
No
Yes, increased

Yes, decreased
- Do you have family members with celiac disease?
Yes
No
-If yes, who?
Parent
Partner
Offspring
Other
-Do you consider yourself compliant with the restrictive measures given by the Government about lock down and social distancing?
Very much
Much
Enough
Little
Not at all
-Are you currently quarantined by the Authorities?
No
Yes, because I have COVID-19-like symptoms
Yes, because I have a confirmed COVID-19 disease
No, I am hospitalized
-Where do you live?
Big city downtown
Suburbs
Small/ medium countryside town
Small/medium seaside town
Isolated house/small village with few hundred residents
- Are the electronic devices in your possession adequate to your needs?
Not at all
A little
Enough
Much
Very much
- Is your income adequate to your needs?
Not at all
A little
Enough
Much
Very much

2. Data about celiac disease

- Year of celiac disease diagnosis
…………..
-Did you undergo upper digestive tract endoscopy for the diagnosis of celiac disease?
Yes
No
- Why did you perform examination in the suspicion of celiac disease?
Because I had symptoms
Because I had a relative with celiac disease
Incidentally, because I had no symptoms
- Were your celiac disease antibodies negative at the last follow-up control?

Yes
No
- In the last year, before the COVID-19 pandemic, have you experienced symptoms that you think are attributable to celiac disease?
Yes
No
- How is your usual adherence to the gluten-free diet?
Very strict
Quite strict, with some transgressions
Not very strict, with several transgressions
Not strict at all

3. Data about gluten-free diet during COVID-19 pandemic

- During the Government lockdown, have you had any difficulties finding gluten-free products dispensed with the voucher?
Yes
No
- If yes, for what reason?
Impossibility to leave my house for the restrictive measures
Impossibility to use the voucher because I am outside my region of residence
Difficulties in reaching the stores
Other
- During the Government lockdown, have you tried homemade recipes with naturally gluten-free ingredients?
Yes, as I usually do
Yes, more than usual
No, I didn't have time
No, I prefer to use ready-made foods
No, I'm not capable
No, I don't like them
No, I'm concerned about possible contaminations in the making
Other
- During the Government lockdown, you have the impression that your adherence to the gluten-free diet is:
Stricter than before
Less strict than before
Unchanged
- If you believe that your adherence to the gluten-free diet in this period is better than before, what do you believe might be the reasons? (more than one answer can be selected)
Not eating away from home
More time to prepare food
No social life/home alone
Taking better care of myself
I don't know
Other
- If you believe that your adherence to the gluten-free diet in this period is worse than before, what do you believe might be the reasons? (more than one answer can be selected)
Less time to prepare food
Boredom / sadness / worries about the general situation
More occasions for transgression
Difficulties in finding gluten-free products
I don't know
Other

3. Care aspects
- During the Government lockdown, have you had symptoms in your opinion attributable to celiac disease?
Yes
No
- During the Government lockdown, who did you consult if you needed health care advice on celiac disease?
Nobody, I didn't need it
Gastroenterologist
Dietician
Nutritionist
Family doctor
Private doctor
Patient association
Friends with celiac disease
Other
- During the Government lockdown, how did you receive health care advice regarding celiac disease?
I didn't need it
Telephone or e-mail contact with my celiac disease center
Telephone or e-mail contact with a patient association
Telephone or e-mail contact with my family doctor
Telephone or e-mail contact with my private doctor
Outpatient visit
Home visit
Other
- During the Government lockdown, did you skip previously scheduled appointments es. checks / exams related to celiac disease?
Yes
No
- Do you think that the restrictive measures have had an impact on the management of your celiac disease?
Yes, for the worse
Yes, for the better
No
- If they had an impact, would you like to tell us how?
………….

<u>Session 4: Questionnaire for parents/caregivers of young subjects (<18 years) with a certified diagnosis of celiac disease</u>

1. Demographic data

- Year of birth of the respondent
………………..
- Nationality of the respondent
……………..
- Province of residence of the respondent
……………..
- Education degree of the respondent
None
Primary school
Junior High School
High School
Bachelor degree

Master degree
- *Are you currently?*
Unemployed
Student
Housewife
Employed
Retired
- *During these days about my job*
I am working from home/smart working
I am regularly going to my workplace
I am going to my workplace less frequently
My job has been suspended
I got fired
I am on maternity leave
I am in layoffs
I am retired
- *How many people live in your household?*
…………
- *Has the number of members in your household changed during the COVID-19 pandemic?*
No
Yes, increased
Yes, decreased
- *In which year was the child / adolescent with celiac disease you will talk about in this questionnaire born?*
…..
- *Are there any other people with celiac disease besides him / her in your family?*
Yes
No
- *If yes, who?*
Parent
Partner
Sibling
Me
Other
- *Do you consider yourself compliant with the restrictive measures given by the Government about lock down and social distancing?*
Very much
Much
Enough
Little
Not at all
- *Are you currently quarantined by the Authorities?*
No
Yes, because I have COVID-19-like symptoms
Yes, because I have a confirmed COVID-19 disease
No, I am hospitalized
- *Where do you live?*
Big city downtown
Suburbs
Small/ medium countryside town
Small/medium seaside town
Isolated house/small village with few hundred residents
- *Are the electronic devices in your possession adequate to your needs?*

Not at all
A little
Enough
Much
Very much
- Is your income adequate to your needs?
Not at all
A little
Enough
Much
Very much

2. Data about celiac disease

- Year of celiac disease diagnosis of your child
…………..
- Did he/she undergo upper digestive tract endoscopy for the diagnosis of celiac disease?
Yes
No
- Why did he/she perform examination in the suspicion of celiac disease?
Because he/she had symptoms
Because he/she had a relative with celiac disease
Incidentally, because he/she had no symptoms
- Were his/her celiac disease antibodies negative at the last follow-up control?
Yes
No
- In the last year, before the COVID-19 pandemic, have he/she experienced symptoms that you think are attributable to celiac disease?
Yes
No
- How is his/her usual adherence to the gluten-free diet?
Very strict
Quite strict, with some transgressions
Not very strict, with several transgressions
Not strict at all

3. Data about gluten-free diet during COVID-19 pandemic

- During the Government lockdown, have you had difficulties in finding gluten-free products dispensed with the voucher?
Yes
No
- If yes, for what reason?
Impossibility to leave my house for the restrictive measures
Impossibility to use the voucher because I am outside my region of residence
Difficulties in reaching the stores
Other
- During the Government lockdown, have you tried homemade recipes with naturally gluten-free ingredients?
Yes, as I usually do
Yes, more than usual
No, I didn't have time
No, I prefer to use ready-made foods

No, I’m not capable
No, my child doesn’t like them
No, I'm concerned about possible contaminations in the making
Other
- During the Government lockdown, you have the impression that your kid’s adherence to the gluten-free diet is:
Stricter than before
Less strict than before
Unchanged
- If you believe that the adherence to the gluten-free diet in this period is better than before, what do you believe may be the reasons? (more than one answer can be selected)
Not eating away from home
More time to prepare food
Taking better care of my child
Higher control over my child
I don’t know
Other
- If you believe that the adherence to the gluten-free diet in this period is worse than before, what do you believe may be the reasons? (more than one answer can be selected)
Less time to prepare food
Boredom / sadness / worries about the general situation
More occasions for transgression
I don’t know
Other

3. Care aspects

- During the Government lockdown, has your child had symptoms in your opinion attributable to celiac disease?
Yes
No
- During the Government lockdown, who did you consult if you needed health care advice on celiac disease for your child?
Nobody, I didn't need it
Gastroenterologist
Dietician
Nutritionist
Family doctor
Private doctor
Patient association
Friends with celiac disease
Other
- During the Government lockdown, how have you received health care advice regarding celiac disease for your child?
Telephone or e-mail contact with my celiac disease center
Telephone or e-mail contact with a patient association
Telephone or e-mail contact with my family doctor
Telephone or e-mail contact with my private doctor
Outpatient visit
Home visit
Other
- During the Government lockdown, has your child skipped skip previously scheduled appointments es. checks / exams related to celiac disease?
Yes
No

- *Do you think that the restrictive measures have had an impact on the management of your child's celiac disease?*

Yes, for the worse

Yes, for the better

No

- *If they had an impact, would you like to tell us how?*

………….
